# Therapeutic manipulation of angiogenesis with miR-27b

**DOI:** 10.1186/s13221-015-0031-1

**Published:** 2015-06-24

**Authors:** Dorina Veliceasa, Dauren Biyashev, Gangjian Qin, Sol Misener, Alexander Roy Mackie, Raj Kishore, Olga V. Volpert

**Affiliations:** Urology Department, Northwestern University Feinberg School of Medicine, Chicago, IL USA; Department of Urology, University of Illinois at Chicago Medical College, Chicago, IL USA; Department of Medicine, Cardiology Division, Northwestern University Feinberg School of Medicine, Chicago, IL USA; Department of Radiology, Northwestern University Feinberg School of Medicine, Chicago, IL USA; Department of Pharmacology, Temple University School of Medicine, Philadelphia, PA USA; Northwestern University, Feinberg Cardiovascular Research Institute, Chicago, IL USA

**Keywords:** miRNA, miR-27b, Therapeutic angiogenesis, Ischemia, Cardiovascular, Cancer

## Abstract

**Background:**

Multiple studies demonstrated pro-angiogenic effects of microRNA (miR)-27b. Its targets include Notch ligand Dll4, Sprouty (Spry)-2, PPARγ and Semaphorin (SEMA) 6A. miR-27 effects in the heart are context-dependent: although it is necessary for ventricular maturation, targeted overexpression in cardiomyocytes causes hypertrophy and dysfunction during development. Despite significant recent advances, therapeutic potential of miR-27b in cardiovascular disease and its effects in adult heart remain unexplored. Here, we assessed the therapeutic potential of miR-27b mimics and inhibitors in rodent models of ischemic disease and cancer.

**Methods:**

We have used a number of models to demonstrate the effects of miR-27b mimicry and inhibition in vivo, including subcutaneous Matrigel plug assay, mouse models of hind limb ischemia and myocardial infarction and subcutaneous Lewis Lung carcinoma.

**Results:**

Using mouse model of myocardial infarction due to the coronary artery ligation, we showed that miR-27b mimic had overall beneficial effects, including increased vascularization, decreased fibrosis and increased ejection fraction. In mouse model of critical limb ischemia, miR-27b mimic also improved tissue re-vascularization and perfusion. In both models, miR-27b mimic clearly decreased macrophage recruitment to the site of hypoxic injury. In contrast, miR-27b increased the recruitment of bone marrow derived cells to the neovasculature, as was shown using mice reconstituted with fluorescence-tagged bone marrow. These effects were due, at least in part, to the decreased expression of Dll4, PPARγ and IL10. In contrast, blocking miR-27b significantly decreased vascularization and reduced growth of subcutaneous tumors and decreased BMDCs recruitment to the tumor vasculature.

**Conclusions:**

Our study demonstrates the utility of manipulating miR-27b levels in the treatment of cardiovascular disease and cancer.

**Electronic supplementary material:**

The online version of this article (doi:10.1186/s13221-015-0031-1) contains supplementary material, which is available to authorized users.

## Background

Angiogenesis is a tightly regulated process, which is critical for organ development and tissue homeostasis in adults [1]. Deregulated angiogenesis underlies numerous pathological conditions and represents an important therapy target [2].

Excessive angiogenesis and vascular leakiness are the hallmarks of cancer and inflammatory disease and several anti-angiogenic agents have been approved for clinical use [3]. Most of them, like neutralizing antibodies (Bevacizumab, Ranizumab) or decoy receptors (VEGF trap, Aflibercept), sequester the key angiogenic factor, Vascular Endothelial Growth Factor (VEGF). The success of anti-angiogenics is most striking in patients with age-related macular degeneration where they normalize the vasculature, curtail vascular leakage/edema and restore visual acuity [4].

Bevacizumab is FDA-approved for metastatic cancers, including colon, lung, ovaries and glioblastoma multiforme of the brain, where it is used with standard chemotherapy and significantly improves the outcomes. However, the use of VEGF-targeting agents is associated with severe systemic toxicities, which stem from its role in cell survival and include severe hypertension and thromboembolism [5], renal failure [6], intestinal perforation [7, 8], and neuropathies [9]. Thus, the need remains for the new, safer anti-angiogenic treatments [10].

On the other hand, pathologic conditions including peripheral arterial disease (PAD) and myocardial infarction (MI), in which ischemia causes irreversible tissue damage could benefit from enhanced angiogenesis and subsequent accelerated reperfusion and improved tissue homeostasis [11]. Gene therapy with angiogenic factors such as VEGF, basic fibroblast growth factor (bFGF) and hepatocyte growth factor (HGF) was very effective in preclinical models of critical limb ischemia (CLI) and MI [12–15]. However, randomized, double blinded placebo controlled clinical studies showed limited or no improvement [16, 17]. Another approach to revascularization of ischemic tissue is represented by stem cell-based therapies, which show benefits in clinical and preclinical studies [18, 19]. However, larger trials are needed to confirm the improvement of the clinically relevant outcomes.

MicroRNA (miRNA) are rapidly emerging as important regulators of all aspects of angiogenesis including vascular sprouting [20, 21], proliferation, survival and migration [21, 22] of the vascular endothelial cells and recruitment of the vascular progenitor cells [23]; miRNA are also involved in maturation of the newly formed vasculature, by controlling angiopoietin expression [24]. Unlike VEGF inhibitors, miRNA provide a more subtle degree of regulation whereby they moderately decrease the expression of multiple target genes involved in the same or related biological processes [25].

Pro-angiogenic function of miR-27b was demonstrated in several studies [26–28]. We have shown previously that miR-27b promotes capillary sprouting and endothelial tip fate by suppressing the Notch ligand, Delta-like ligand 4 (Dll4) and the negative regulator of branching, Sprouty-2 (Spry-2) [26]. Others have shown that miR-27b also inhibits Spry-2 and repulsive signaling by Semaphorin 6A [27, 28].

Here, we provide experimental evidence that therapeutic manipulation of miR-27b can be beneficial in angiogenesis-dependent disease. Limited local application of miR-27b mimic was sufficient to restore angiogenesis and improve tissue function in two models of ischemic disease, hind limb ischemia, which mimics CLI, and coronary artery ligation, a MI model. Importantly, miR-27b increased the recruitment of bone marrow derived cells (BMDCs) to the neovasculature, as was shown in mice reconstituted with GFP-tagged bone marrow. In both models, miR-27b significantly decreased inflammation as was evidenced by decreased macrophage recruitment at the site of ischemic injury. These effects were due, at least in part, to the decreased expression of Notch ligand Dll4, PPARγ and of inflammatory cytokines. In a contrasting approach, blocking miR-27b by systemic administration of an anti-miR oligonucleotide significantly decreased microvacular density (MVD) and delayed the growth of highly aggressive subcutaneous tumors (Levis Lung Carcinoma, LLC-1). Surprisingly, treatment with anti-miR-27b also reduced the recruitment and activation of tumor-associated macrophages (TAMs). Together, our results demonstrate potential utility of manipulating miR-27b levels in the treatment of cardiovascular disease and cancer as well as associated inflammatory processes and indicate several molecular mechanisms involved.

## Materials and methods

### Cells and reagents

Lewis lung carcinoma (LLC1) and RAW 264.7 mouse macrophage cell lines (ATCC, Manassis, VA) were cultured in Dulbecco’s Modified Eagle’s Medium (DMEM) supplemented with 10 % FBS and 1× penicillin/streptomycin.

MirVana brand miRNA mimics (double-stranded oligonucleotides, with star strand inactivated by chemical modifications for maximal specificity) included negative control (NC) and mmu-miR-27b and miR-27b inhibitor (antagomir). All were purchased from Life Technologies (Grand Island, NY) and used with transfection reagent in vivo-JetPEI (Polyplus-transfection, New York, NY).

### Experimental animals

FVB mice (10–12 weeks old, Jackson Laboratory, Bar Harbor, Maine), immune-compromised athymic nude mice (hsd:athymic nude-foxn1), immune-competent C57/Bl6 mice (4–6 weeks old, Harlan, Madison, WI) and transgenic mice C57BL/6-Tg (CAG-EGFP) 1Osb/J (Jackson Laboratory, Bar Harbor, Maine) were housed at Northwestern University Center for Comparative Medicine and treated in accordance with the NIH guidelines and protocols approved by Northwestern University Animal Care and Use Committee (ACUC ASP 2012–2864).

### Matrigel plug assay

Matrigel assay was performed as described previously [29]. Ice-cold matrigel (BD Biosciences, San Jose, CA) was supplemented with Heparin 60U/ml, with or without VEGF (200 ng/ml). miR27b and NC mimic (20 μg/mouse) were formulated with in vivo-JetPEI (PolyPlus Transfection, France) in 5 % glucose, according to manufacturer’s instructions, and added, where indicated. Matrigel (0.4 ml/mouse) was injected subcutaneously in the median abdominal area of anesthetized nude mice and allowed to solidify. After 10 days, the plugs were excised and snap-frozen for further analysis.

### Induction of hind-limb ischemia (HLI)

Ischemia was induced surgically as described previously [30]. FVB mice (10–12-weeks old) were anesthetized with Avertin (tribromoethanol, 250 mg/kg, Sigma, St. Louis, MO). Ischemia was induced by ligation of the femoral artery at the site of bifurcation, the epigastric artery and at the bifurcation of the femoral artery, and the popliteal artery followed by the removal of the entire femoral artery. The animals were followed for 14 days with visual inspection and laser Doppler imaging [31]. On day 14, the animals were euthanized and the quadriceps and gastrocnemius muscle harvested and frozen in TissueTek OCT compound (Fisher).

### Laser doppler imaging (LDI)

Blood flow in the hind legs of anesthetized animals was measured under standardized conditions, with Laser Doppler Imager (Moor Instruments, UK). The mice were kept on a heating pad (37 °C) to minimize variation between flow measurements. The perfusion ratio between ligated and intact leg was calculated for each time point.

### Induction of myocardial infarction by coronary artery ligation (CLI)

C57/Bl6 mice (10 weeks old) were anesthetized with Avertin (250 mg/kg) and orally intubated with a 22-gauge catheter, for artificial ventilation with a respirator (Harvard Apparatus). A left intercostal thoracotomy was performed and the ribs were retracted with 5–0 polypropylene sutures to open the pericardium. The left anterior descending (LAD) branch of the left coronary artery was ligated under dissecting microscope (Leika), distal to the bifurcation between LAD and diagonal branch with 8–0 polypropylene sutures. After positive end-expiratory pressure was applied to fully inflate the lung, the chest was closed with 7–0 polypropylene sutures [32].

### Physiological assessment of left-ventricular function

Trans-thoracic 2-dimensional measurements were performed using a high-resolution echocardiography system (VEVO 770™, VisualSonics Inc., Toronto, Canada) equipped with a 30-MHz transducer. Measurements were taken14 and 28 days post-MI on mice anesthetized with a mixture of 1.5 % isoflurane and oxygen (1 L/min). M-mode tracings were used to measure LV wall thickness, end-systolic diameter (LVESD), and end-diastolic diameter (LVEDD). Systolic and diastolic left-ventricular areas were determined by M-mode in long-axis configuration and fractional shortening (FS) was measured at the mid-ventricular level. The left-ventricular chamber volumes in diastole and systole were derived from their respective measured 2D areas using a LV volume algorithm within the Vevo770 echo software. Cardiac ejection fraction was determined offline by the equation: [EF = (Diastolic Volume − Systolic Volume/Diastolic Volume) × 100 %].

### Irradiation of mice and bone marrow transplantation

C57/Bl6 female mice (6–8 weeks old) were subjected to sub-lethal whole body irradiation (500 rads/mouse). Bone marrow was isolated by flushing the femurs and tibias of isogenic mice, Tg (CAG-EGFP) 13OsbLeySopj, with a 26½-gauge needle and passing through a 70-um mesh sterile filter. After 1-day rest, the mice were transplanted with the bone marrow cells (3 × 10^6^ cells/mouse in 200 μl), by tail vein injection through a 27½-gauge needle. The recipient mice were treated with 1.6 mg/ml Sulphametoxazole/Trimethoprim (Bristol-Meyers-Squibb, New York, NY) in drinking water and allowed 8 weeks recovery.

### Tumor implantation

C67Bl6 mice (12–14 weeks) reconstituted with the GFP-tagged bone marrow were injected subcutaneously in the right hindquarters with of 0.5 × 10^6^ Lewis Lung Carcinoma cells (LLC1, ATCC, Manassis, VA) in 0.1 mL PBS. When tumors reached 5 mm in diameter, animals were randomized into groups (*n* = 5) and treated with miR-27b mimic, inhibitor and negative control complexes prepared in sterile 5 % glucose using in vivo-jetPEI (PolyPlus Transfection, France).

### Treatment of mice

After hind limb surgery or MI, mice were randomized into groups (*n* ≥ 6). In HLI model, mice were treated by intramuscular injections of miR27b and NC mimics into the quadriceps muscle (10 μg/mouse/day, 2 injections in a total of 50 μl, via 27-gauge needle). miRNA were formulated with jetPEI in 5 % glucose. For the myocardial infarction (MI) model, miR-27b and NC mimics formulated as above were administered at the time of surgery by intracardiac injections (10 μg/mouse, two 10 μl injections), using a 10 μl Hamilton syringe with a 30-gauge needle. Mice with LLC tumors (5–7 mm diameter) were randomized into groups (*n* = 5) and treated with intraperitoneal injections of NC, miR-27b mimic or inhibitor, formulated as above (100 μg/100 μl/mouse). At the endpoint mice were sacrificed and tissues harvested for analysis.

### Immunohistochemistry

All antibodies and dilutions used for this procedure are listed in Table 1 below. To detect blood vessels, 5-μm thick matrigel plug or tumor cryosections were stained with rat anti-mouse CD31 antibody (1:100, BD Pharmingen, San Jose, CA) followed by donkey anti-rat Rhodamine Red-X conjugated secondary antibodies (1:1000, Jackson Immunoresearch). Dll4 in frozen sections was detected using rabbit anti-mouse antibody (1:100, Abcam, Cambridge, MA) followed by goat anti-rabbit Alexa Fluor 488 conjugate (Jackson Immunoresearch, West Grove, PA). The nuclei were visualized with 4′,6-diamidine-2-phenyl indole (DAPI). Macrophages were detected with rat anti-mouse antibody against F4/80 cell surface antigen (1:100, Affymetrix, Santa Clara, CA) followed by donkey anti-rat Rhodamine conjugate (Jackson Immunoresearch). GFP on frozen sections was visualized with rabbit anti-mouse antibody (1:1000, Abcam, Cambridge, MA) and donkey anti-rabbit Alexa Fluor 488 conjugate (Life Technologies, Grand Island, NY). Digital images of immunostained sections were obtained with Nikon Diaphot 2000 epifluorescence. Paraffin-embedded tissues were processed and stained at Northwestern University Pathology Core to detect endothelial cell marker CD31, smooth muscle actin (SMA), Collagen (Col) I, macrophage marker F4/80 and with Masson Trichrome reagent to detect fibrotic areas.

**Table 1:** Antibodies used in the study (immunostaining analyses)

Antibody	Type	Vendor	Cat. no.	Dilution
CD31 (rat anti-mouse)	1°	BD Pharmingen, San Jose, CA	553370	10^−2^
Dll4 (rabbit anti-mouse)	1°	Abcam, Cambridge, MA	ab7280	10^−2^
F4/80 surface antigen (rat anti-mouse)	1°	Affymetrix, Santa Clara, CA	14-4801	10^−2^
Green Fluorescent Protein (rabbit anti-mouse)	1°	Abcam, Cambridge, MA	ab6556	10^−3^
Smooth Muscle Actin (rabbit anti-mouse)	1°	Abcam, Cambridge, MA	ab5694	10^−2^
Collagen I (rabbit anti-mouse)	1°	Abcam, Cambridge, MA	ab34710	10^−2^
Donkey anti-rat, RR-X conjugate	2°	Jackson Immuno Research, West Grove, PA	712-295-153	10^−2^
Goat anti-rabbit, Alexa Fluor 488 conjugate	2°	Jackson Immuno Research, West Grove, PA	111545-144	10^−2^
Donkey anti-rabbit, Alexa Fluor 488 conjugate	2°	Life Technologies, Grand Island, NY	A-21206	1.5 × 10^−2^

### Electroporation of cells

RAW 264.7 macrophages were transfected by electroporation using D-032 Nucleofector device with Amaxa Cell Line Nucleofector kit V (Lonza, Walkersville, MD) following manufacturer’s instructions. For every 2 × 10^6^ 30 nM miR-27b mimic, inhibitor, NC RNAi or 2 μg GFP plasmid (transfection control) were in added. After electroporation, the cells were grown 48 h and total RNA for RT-PCR was extracted as described above.

### Morphometric and statistical analysis

Images were analyzed in a blinded fashion with NIS-Elements BR software (Nikon Inc., USA). GraphPad Prism software (Version 5.01, La Jolla, USA) was used for statistical analyses. For quantitative traits, all datasets were subjected to descriptive statistical analysis to establish normality, using Kolmogorov-Smirnov and D’Agostino-Pearson normality tests. Normally distributed datasets were compared pairwise or as a group using 2-tailed Student’s *t* test or Tukey’s multiple comparisons test. Non-normally distributed datasets were compared using Mann–Whitney test for single-time measurements. For multiple time points, two-way ANOVA was used followed by Bonferroni posttest for normally distributed data and Kruskal-Wallis for non-normal distribution. *P* < 0.05 was considered significant.

### Quantitative real-time polymerase chain reaction (Q-PCR)

Total RNA was extracted with RNeasy isolation kit (Qiagen, Valencia, CA), per manufacturer’s instructions. Dll4, Spry-2, Col I and SMA were amplified with Sso Advanced SYBR Green Supermix kit (Bio-Rad, Hercules, CA) with the primers specific for mouse genes (Table 2).

**Table 2:** Primers used in the study

Gene	Direction	Sequence (5′–3′)
Collagen 1	Forward	TCTGACTGGAAGAGCGGAGAG
	Reverse	GGCACAGACGGCTGAGTAGG
Dll4	Forward	TCGAAATGGTGGCAGCTGTAAGGA
	Reverse	ATGCTCACAGTGCTGGCCATAGTA
IL10	Forward	CTCCAAGACCAAGGTGTCTAC
	Reverse	GGAGTCCAGCAGACTCAATAC
IL12	Forward	GGGAGAAGCAGACCCTTACA
	Reverse	TTCAGGCGGAGCTCAGATAG
PPARγ	Forward	CTGGCCTCCCTGATGAATAAAG
	Reverse	AGGCTCCATAAAGTCACCAAAG
SPRY-2	Forward	ACTGCTCCAATGACGATGAGGACA
	Reverse	CCTGGCACAATTTAAGGCAACCCT
SMA	forward	TGACAGAGGCACCACTGAACC
	reverse	TCCAGAGTCCAGCACAATACC AGT

## Results

### miR-27b mimicry promoted *de novo* angiogenesis and BMDC recruitment

**Fig. 1:**
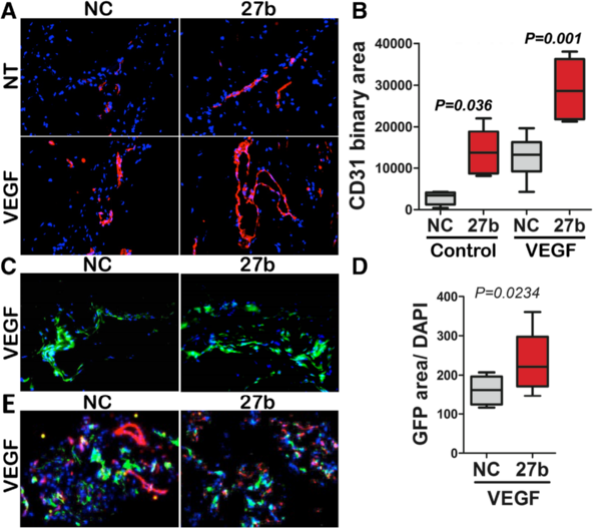
miR-27b augments VEGF-induced angiogenesis and recruitment of the bone marrow -derived cells. Extracellular matrix (Matrigel) supplemented with Heparin (60 U/ml) to retain growth factors, was injected in mice to generate subcutaneous gel plugs. VEGF, miR-27b mimics (27b) or negative control RNAi (NC), were added to Matrigel as indicated. The plugs were harvested and processed for analysis after 10 days. (**a**) Sections of cryopreserved Matrigel plugs were stained for the endothelial marker, CD31 (*red*) and cell nuclei visualized with DAPI (*blue*). (**b**) Fluorescence images (×20, a minimum of 10 per condition) were taken and CD31-positive vascular area measured using Elements software (Nikon). The data is presented as box plot with whiskers; median values are shown. *P* value was calculated using Kruskal-Wallis test. Note increased vascularization in the presence of miR-27b. (**c**) Mice were lethally irradiated and reconstituted with bone marrow (BM) harvested from mice with ubiquitous expression of actin-GFP transgene. After 6-week recovery, mice were used in Matrigel plug assay for angiogenesis, as in (**a**). GFP-expressing BM-derived cells (BMDCs) are shown in *green*. (**d**) The recruitment of the GFP-positive BMDCs to the Matrigel (green fluorescence area) was measured using Elements software. The measurements from at least eight ×20 fields per condition are graphed as box plot with whiskers; median values are shown. *P* value is calculated using Wilcoxon Signed Rank test. Note elevated BMDC recruitment in response to miR-27b. (**e**) Dual immunofluorescence for the endothelial marker, CD31 (*red*) and GFP-positive BMDCs (*green*). Note minimal co-localization between CD31 and GFP (*yellow*)

We have used in vivo assay for angiogenesis in subcutaneous Matrigel plugs [29]. Adding miR-27b mimic to the Matrigel clearly increased the number of CD31-positive vascular structures at baseline, in the absence of pro-angiogenic VEGF and significantly augmented angiogenic response to VEGF (Fig. 1a, b).

Neovasculature can be assembled from the endothelial cells formed by local proliferation of pre-existing endothelium, or by incorporation of the circulating precursors that originate in the bone marrow (bone marrow derived cells, BMDCs) [33]. To assess the contribution of the BMDCs to the pro-angiogenic function of miR-27b we used Matrigel plug assay in mice transplanted with GFP-tagged bone marrow from Tg (CAG-EGFP) 13OsbLeySopj mice. miR-27b significantly increased recruitment of GFP-positive BMDCs to the site of VEGF-induced neovascularization (Fig. 1c, d). However, in contrast with previously published results [34], the majority of the GFP-positive BMDCs cells neither assumed endothelial fate nor were incorporated in the neovessels, as was evidenced by the lack of co-localization between GFP and the endothelial marker CD31 (Fig. 1e).

### miR-27b enhanced angiogenesis and improves perfusion in the ischemic hind limb

**Fig. 2:**
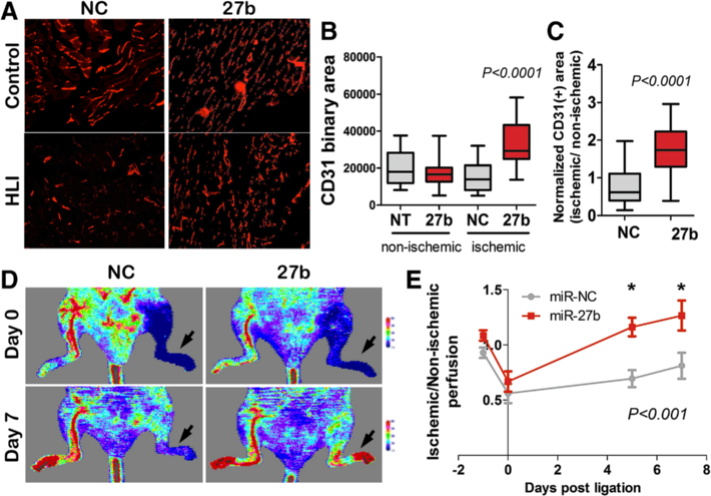
miR-27b improves perfusion in murine model of critical limb ischemia. Hind limb ischemia (HLI) was induced in FVB mice by surgical ligation of the femoral arteries on day 0. Laser Doppler scans (LDS) have been performed for each animal prior to surgery (day −1), after ligation (day 0) to confirm the onset of ischemia, and on days 5 and 7, to assess recovery. On day 0, mice were randomly assigned to groups (*n* = 5) and injected three times with negative control (RNAi) or miR-27b mimic (10 μg/injection/day, days 0–2; NC and 27b, respectively). Tissues were harvested and processed on day 14. (**a**) At endpoint (day 14) calf muscles were harvested and snap-frozen for analysis. Five-μm crosswise sections were stained for the endothelial marker, CD31 (*red*), to assess microvessel density (MVD). Representative photomicrographs are shown. (**b**, **c**) CD31-positive vascular area was measured using Elements software (Nikon) on the images of at least nine fields from three independent sections (×20 objective). The data are presented as box plots with whiskers. *P* values were calculated using Tukey’s Multiple Comparisons test (**b**) Note increased vascular area in ischemic tissue upon miR-27 treatment (27b) compared to control (NC). (**c**). To correct for the baseline MVD in each animal, the ratio between ischemic (HLI) and non-ischemic (control) limb was calculated for miR-27b and control-treated animals. *P* value was calculated using one-sample *t* test. (**d**) The representative LDS images for days 0 and 7. Color scale for perfusion range is shown, with *blue* indicating impeded perfusion. Ischemic legs are indicated by *arrows*. (**e**) The perfusion was quantified and normalized against control (intact) limb; mean values and S.E.M. were calculated for each time point. *P* values were calculated by the Two-way RM ANOVA and Bonferroni posttest. Note progressive improvement of blood flow in ischemic limbs in miR-27b group (27b) compared to control treated group (NC)

We then tested whether miR-27b similarly accelerates angiogenesis in the ischemic muscle. Hind Limb Ischemia (HLI) in FVB mice was induced by femoral artery ligation. After surgery, the mice received intramuscular (IM) injections of miR-27b mimic or negative control (non-silencing RNAi) for three consecutive days. Treatment with miR-27b mimic significantly improved tissue vascularization, as was shown by immunofluorescence for the endothelial marker, CD31 (Fig. 2a-c). In agreement, miR-27b mimic caused a statistically significant improvement in tissue perfusion by day seven post-ligation, as measured by Doppler Laser Imaging (DLI) (Fig. 2d, e).

### miR-27b preserved angiogenesis and protected tissue function in the infarcted heart

**Fig. 3:**
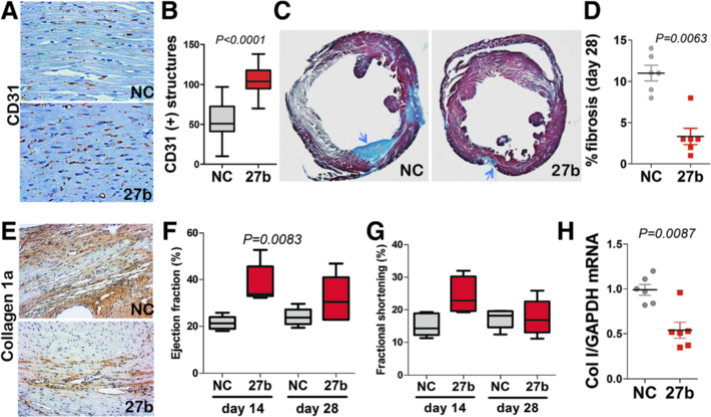
miR-27b is cardioprotective and pro-angiogenic in a mouse model of myocardial infarction (MI). MI was induced in C57Bl6 mice by ligating left coronary artery. Negative control RNAi (NC) or miR-27b mimics were formulated with Jet-PEI and injected into cardiac muscle at the time of surgery (10 μg/mouse). Transthoracic echocardiography was performed 14 and 28 days post-surgery and the animals were sacrificed on day 28. (**a**, **b**) Immunohistochemistry (**a**) and quantitative analysis (**b**) of the endothelial marker, CD31, in the hearts of mice treated with NC or miR-27b. The data is presented as boxplot with whiskers. *P* value was calculated by Kruskal-Wallis method. (**c**, **d**) Apical heart sections were assessed for fibrosis using Masson-Trichrome staining. (**c**) Representative images of the hearts treated with NC and miR-27b mimic (27b), respectively. *Arrows* point to the areas of fibrosis (*blue*). (**d**) Quantitative analysis of fibrotic areas was performed using Nikon Elements software; data is shown as dot plot with whiskers. *P* value was calculated by Kruskal-Wallis method. (**e**) Five-μm horizontal apical sections of the hearts treated as indicated were stained for collagen 1, for additional assessment of fibrosis. (**f**) Total RNA was extracted from cryopreserved tissue isolated from the infarcted areas and expression of Collagen I (Col I) measured by real-time RT-PCR with GAPDH served as an internal control. Data is presented as dot plot with whiskers. *P* value was determined using Mann–Whitney (Wilcoxon) test. (**g**, **h**) Left ventricular cardiac function, including ejection fraction (**g**) and fractional shortening (**h**) were measured using images obtained by two-dimensional M-mode echocardiography; statistical analysis was performed using Kruskal-Wallis test. Note a significant increase in the ejection fraction at day 14 in the hearts treated with miR-27b

In the mouse model of myocardial infarction (MI) caused by coronary artery ligation (CLI), a single application of miR-27b mimic had strong protective effect on the microvasculature as was determined by IHC for CD31, with 1.6-fold higher MVD in the infarcted area compared to control group (Fig. 3a, b). Improved vascularization was associated with a significant degree of tissue protection in the animals treated with miR-27b mimic. MiR-27b significantly alleviated fibrosis, as was measured by Masson Trichrome staining (Fig. 3c, d), and Collagen 1 deposition and expression as was determined by IHC, and RT-PCR, respectively (Fig. 3e, h). Similar trend was observed for smooth muscle actin (Additional file 1: Figure S1). Importantly, these changes caused by miR-27b mimicry resulted in improved cardiac function, with an approximately 2-fold increase in ejection fraction over control by day 14 (Fig. 3f) and a trend towards increased fractional shortening (Fig. 3g).

### miR-27b inhibitor suppresses cancer growth and angiogenesis

**Fig. 4:**
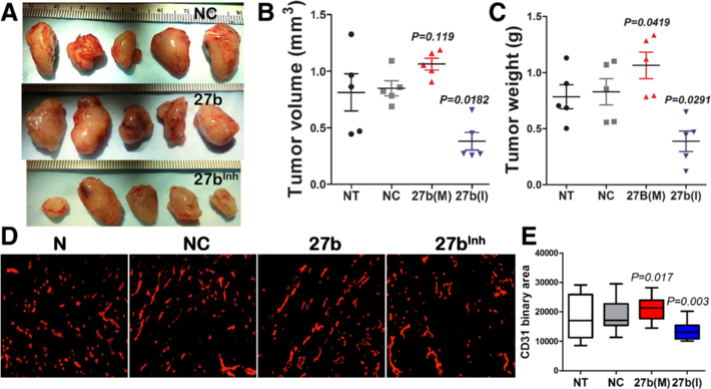
miR-27b inhibition halts tumor growth and angiogenesis. Mice were implanted with subcutaneous LLC tumors in the right flank and tumor growth was measured daily. When tumors reached 5–6 mm in diameter, the animals were treated every 2 days with intraperitoneal injections of NC RNAi, miR-27b mimic 27b(M) or inhibitor, 27b(I) at 100 μg per mouse per injection (*n* = 5). After 1 week, the mice were sacrificed, tumors harvested, weighted and cryopreserved for further analyses. (**a**) Gross appearance of the tumors. (**b**, **c**) Tumor volume (**b**) and weight (**c**) at the endpoint. Data is presented as dot plot with whiskers and median values are shown. *P* values are calculated using Kruskal-Wallis method. (**d**, **e**) Five-μm cryosections were stained for the endothelial marker, CD31 (D), and MVD measured as total CD31-positive area (**e**). The results are presented as box plot with whiskers and *P* value calculated using Wilcoxon Signed Rank method. Note decreased MVD when miR-27b is inhibited, 27b(I). NT, no treatment; NC, negative control; 27b(M), miR-27b mimic; 27b(I), miR-27b inhibitor

Angiogenesis is a rate-limiting factor in tumor progression. We therefore examined the effect of miR-27b inhibitor on tumor angiogenesis and associated growth rates. We used Lewis Lung carcinoma, a particularly aggressive mouse tumor cell line, in a syngeneic subcutaneous model. Systemic treatment with miR-27b inhibitor, 27b(I) at 5 mg/kg caused a significant reduction of tumor weight at the endpoint (Fig. 4a-c, Additional file 1: Figure S5). In contrast, miR-27b mimic, 27b(M) had only moderate, albeit statistically significant effect on tumor growth compared to control groups (untreated mice and mice treated with control RNAi). In agreement, treatment with anti-miR-27b significantly reduced tumor MVD, while miR-27b had only modest effect on already robust angiogenesis in the LLC tumors, (Fig. 4d, e).

### miR-27b manipulations alter Dll4 expression in ischemic and tumor tissue

**Fig. 5:**
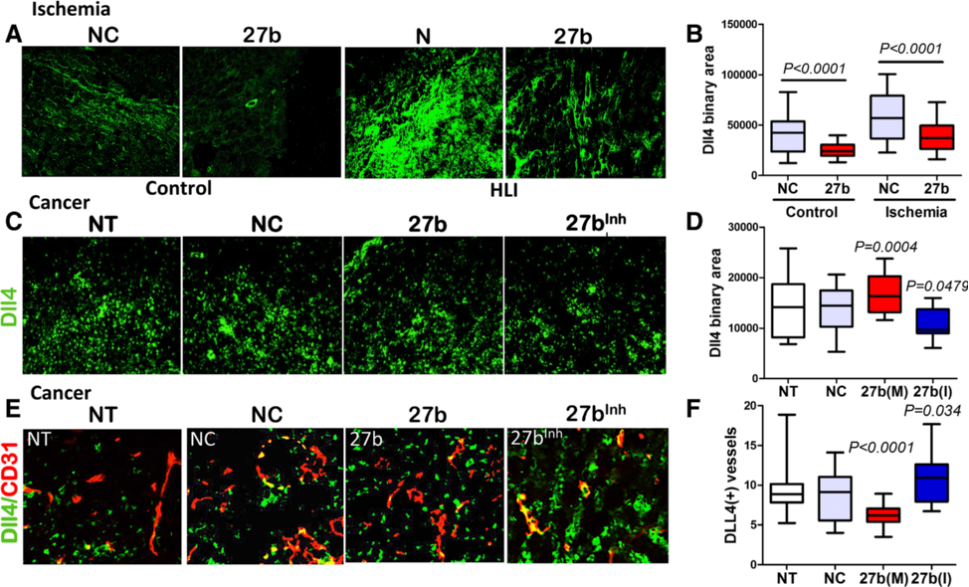
miR-27b controls of Dll4 in ischemic tissue and in vascular endothelium. Five-μm crosswise tissue sections of calf muscle or tumor tissue were stained for Dll4, a miR-27b target (*green*). (**a**) Representative photomicrographs of Dll4 immunostaining of the calf muscle sections. (**b**) Total Dll4 positive area was quantified and adjustment made for fluorescence intensity using Elements software (Nikon). Note increased Dll4 immunostaining under ischemic conditions in animals treated with control RNAi (NC), and significant attenuation by miR-27b (27b). The data are presented as box plot with whiskers. Median values are shown. Statistical significance and *P* values were determined using Wilcoxon Signed Rank test. (**c**, **d**) Sections of tumors from untreated animals, animals treated with control RNAi (NC), miR-27b mimic (M) or inhibitor (I) were stained for Dll4 (green) and total immunofluorescence measured in at least 8 sections/condition using Nikon Elements software. Data is presented as box plot with whiskers and mean values are shown. Statistical significance (*P* values) was determined by Wilcoxon test as above. (**e**) Dual immunofluorescence for Dll4 (*green*) and CD31 (*red*), to determine vascular-specific expression of Dll4. Dual positivity appears as *yellow*. (**f**) Vascular-specific Dll4 expression (a number of Dll4-positive capillary structures/field) was measured using Nikon Elements software and statistical significance calculated as above. *P* values are shown

Previous studies from our group implicated the suppression of Notch ligand, Dll4 in the pro-angiogenic action of miR-27b [26]. In the endothelial cells, Notch signaling interferes with tip cell fate [35]: therefore suppression of Dll4 with miR-27b mimic should allow tip cell fate and increases vascular sprouting, and miR-27b interference should augment Dll4 and inhibit vascular sprouts. This model has been previously confirmed in zebrafish studies. We have ascertained the appropriate increase and decrease of mature miR-27b levels in the target tissues of animals treated with miR-27b mimic and inhibitor, respectively, by real-time PCR (Additional file 1: Figures S2 and S5). IF staining showed elevated Dll4 levels in ischemic calf muscle, which was significantly reduced in the presence of miR-27b mimic (Fig. 5a, b). Similar decrease in Dll4 mRNA was observed by real-time PCR in cardiac tissue of mice treated with miR-27b mimic (Additional file 1: Figure S3). Spry-2, another known target of miR-27b was not significantly altered (Additional file 1: Figure S4).

In subcutaneous LLC tumors, miR-27b had disparate effects on the total and vascular Dll4 expression. LLC tumors showed high baseline levels of Dll4 as was measured by immunofluorescence, and these levels were modestly but significantly increased by 27b(M) and moderately reduced in response to 27b(I) (Fig. 5d, e). In contrast, co-localization analysis with the vascular endothelial marker, CD31, showed a significant reduction of Dll4 specifically on tumor vasculature caused by miR-27b mimic; in agreement, miR-27b inhibitor clearly augmented Dll4 expression by the tumor vasculature (Fig. 5f, g). The difference in the regulation of Dll4 in the tumor cells and vascular endothelium could be due to the tissue context, where miR-27b could cooperate with RNA binding protein(s), such as HuR [36–38] causing mRNA stabilization and translational activation.

### miR-27b alters macrophage infiltration in ischemic and tumor tissues

**Fig. 6:**
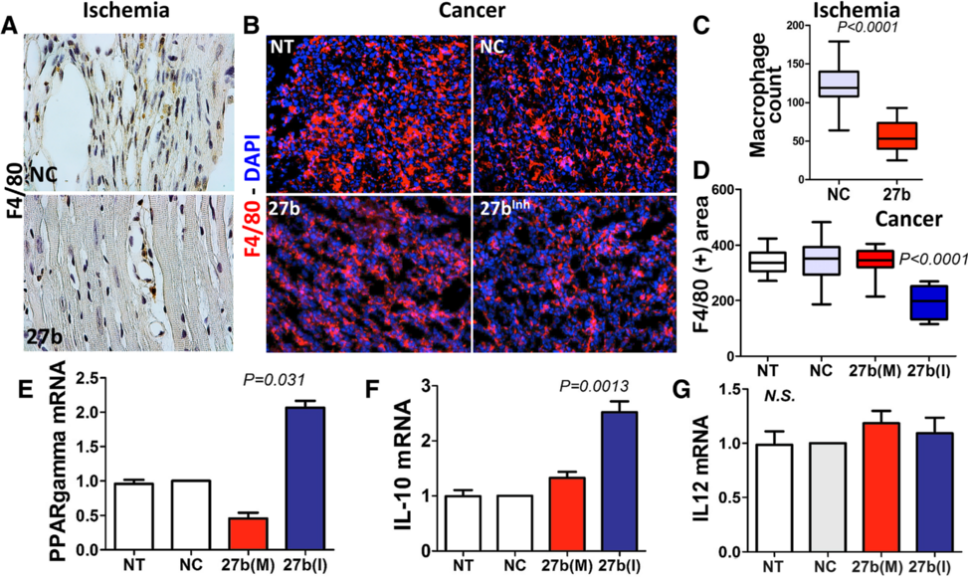
miR-27b regulates inflammatory response in ischemic and tumor tissue. (**a**, **b**) IHC and IF for F4/80 of tissue sections harvested from HLI model (**a**) and LLC tumors treated as indicated. Cell nuclei are visualized with DAPI (*blue*). (**c**-**d**) quantification of the cells positive for macrophage marker, F4/80, (**c**) Macrophages were counted using ImageJ software (National Institutes of Health). Data is presented as box plot with whiskers and *P* values calculated using Mann–Whitney method. (**d**) Macrophages were counted using “object count” function of Nikon Elements software and statistical analysis performed using Wilcoxon test as above. (**e-g**) Cultured macrophages (RAW 264.7) were electroporated with negative control RNAi, miR-27b mimic (M) and inhibitor (I). Gene expression was measured by real-time PCR with L19 and beta macroglobulin as internal controls. PPARγ (**e**), a miR-27b target, and inflammatory chemokines IL-10 (**f**) and IL-12 (**g**) were assessed by real-time RT-PCR and normalized against untreated control (*NT* no treatment). The results of three independent experiments are pooled together. Statistical significance was assessed by Wilcoxon Signed Rank test, as above

miR-27b was previously implicated in the regulation of macrophage responses [39]. We therefore analyzed macrophage infiltration in ischemic and tumor tissues where miR-27b was added (mimic) or blocked (anti-miR). miR-27b significantly attenuated macrophage infiltration to the infarcted area, as was evidenced by IHC for macrophage marker, F4/80 (Fig. 6a). In LLC tumors F4/80, immunofluorescence showed no changes in macrophage infiltration in response to 27b(M). Surprisingly, there was a significant decrease in F4/80 positivity upon treatment with 27b(I).

In LPS-treated macrophages, miR-27b was previously shown to target PPARγ and alter the downstream regulation of inflammatory cytokines [39]. In agreement, electroporation of RAW 264.7 macrophages with 27b(M) caused an approximately 2-fold reduction of PPARγ mRNA (Fig. 6e). On the other hand, treatment with 27b(I) caused a robust increase of PPARγ message (Fig. 6e), which was consistent with decreased macrophage migration/recruitment to the tumor (Fig. 6b, d). Importantly, miR-27b blockade also altered macrophage polarization, which was reflected by the alterations in relative abundance of the M1 and M2 markers, IL12 and IL10 (Fig. 6g, f).

## Discussion

Therapeutic manipulation of angiogenesis in cancer and cardiovascular disease has been explored in pre-clinical models with considerable success. However, toxic effects of current anti-angiogenic therapies necessitate the development of safer options [40], and the development of pro-angiogenic therapies is still in progress [41–43]. miRNA regulators of angiogenesis present an attractive therapeutic option, because they act via relatively subtle regulation of multiple target genes within the same or related pathways [44, 45]. Such a mechanism is more likely to yield sufficient therapeutic benefits with lesser toxicities, because each single target is not fully inactivated. Here, we explore the possibility of therapeutic manipulation of angiogenesis by using mimicry or inactivation, as appropriate, of a pro-angiogenic microRNA, miR-27b.

The role of miR-27b in the regulation of angiogenesis was discovered by several groups [26–28] and its angiogenesis-related targets identified as Dll4 [26], Sema 6A and Sprouty-2 [26, 28, 46]. Silencing of miR-27b leads to decreased VEGF signaling and sprouting angiogenesis in vitro and in vivo (reviewed in [47]). Several studies point to its role in cardiovascular development where it is required for early venous specification of the vascular endothelium [26] and contributes to left ventricular maturation [48] and cardiac hypertrophy [49–51]. It is regulated by pulsatile shear stress suggesting a role in vascular homeostasis. In agreement, simultaneous knockdown of miR-23 and miR-27b in the miR-23/27/24 cluster attenuated neonatal retinal angiogenesis as well as laser-induced choroidal neovascularization [28]. However, miR-27b is also targeted by TGF-β [52] and in vivo overexpression results suggest potential harmful effects in adult heart. Indeed, miR-27b silencing in pressure overload model attenuated hypertrophy, by restoring the expression of its other target, PPARγ [51].

Interestingly, miR-27b deficit could contribute to PAD indirectly, via lipid metabolism. Studies support the role of miR-27b as cholesterol hub in the liver [53]. Hepatic miR-27b is increased in response to hyperlipidemia and, in turn, regulates several key genes in control of lipid metabolism (Angptl3, Gpam) [54]. Moreover, hepatic miR-27b is downregulated and its targets elevated in mouse models of dyslipidemia/atherosclerosis [54]. In addition, miR-27b is decreased in differentiating adipocytes and inhibits their proliferation and accumulation via PPARγ/RXRα controlled pathways [55, 56]. This function of miR-27b could indirectly benefit PAD patients.

In our short-term study, we observed increased vascularization of the muscles of the heart and extremities subjected to ischemic stress, concomitant with preserved tissue integrity and function, suggestive of the therapeutic utility of miR-27b for acute treatment of critical limb ischemia and myocardial infarction. There are similar findings regarding other members of the miR-23/27/24 cluster distinct from miR-27b [57]. It is unclear, however, whether long-term application of miR-27b and/or other miRNA from this cluster could be harmful for cardiomyocytes and require additional targeting to the vascular endothelium, or if there would be long-term beneficial effects on PAD via attenuated lipid metabolism and fat tissue development.

Earlier studies have demonstrated the ability of miR-27b to enhance/restore angiogenic function of the BMDCs in diabetic mice (Db/Db), causing accelerated wound healing [58]. Similarly, we observed the increased recruitment of GFP-tagged bone marrow derived cells to the vasculature. However, the lack of conversion of myeloid BMDCS to the endothelial (CD31-positive) phenotype in vivo suggests that the majority of BMDCs recruited in response to miR-27b treatment have an accessory role where they serve as the source of pro-angiogenic and/or survival factors for the neovasculature.

In cancer, the role of miR-27b is controversial, with studies lending support to its tumor-promoting and tumor-suppressive roles. miR-27b has been identified as a classical oncomir in cell-based assays [59] and its elevated expression is associated with poor prognosis, chemoresistance and metastasis in carcinomas of the ovary and the breast [60] and in castrate-resistant prostate cancer [61]. miR-27b is also upregulated in glioma where it promotes tumor growth and invasion. Consistent with pro-oncogenic role, it is upregulated by Her2 oncogene and by epidermal growth factor [62] and repressed by Ink4A [63, 64] in breast and pancreatic cancer. On the other hand, the 23/27b cluster and miR-27b are downregulated and ascribed anti-tumorigenic roles in the cell lines derived from the tumors of bladder, prostate and colon [39, 61, 65, 66]. Thus the role of miR-27b in cancer progression is likely context-dependent. Our findings suggest that miR-27b has different effects on tumor cells and the cells of tumor microenvironment as is evidenced by its disparate effect on Dll4 expression in the tumor and tumor vasculature. Our findings suggest that the use of miR-27b inhibitors may be feasible in the context of personalized of cancer patients care.

Previous studies indicate that miR-27b contributes towards inflammatory processes by destabilization of PPARγ mRNA and protein [39]. On the other hand, it can block inflammatory responses by targeting NF-κB [67] or TGF-β target, Gremlin-1 [52]. In contrast with published studies, PPARγ expression in cultured macrophages was only modestly affected by mir-27b mimicry, however, it was significantly upregulated by miR-27b inhibitor. On the other hand, miR-27b altered cytokine profile of the cultured macrophages with increased IL-10 and decreased IL-12 levels suggestive of alternative (non-inflammatory) mode of activation. These findings suggest that miR-27 inhibitors may have beneficial effects in cancer setting by blocking the recruitment and activation of tumor-associated macrophages. The reduced inflammatory infiltrates in ischemic tissue may be secondary to anti-fibrotic action of miR-27b. Alternatively, they could be due to the down-modulation of the pro-inflammatory NF-κB. Since miR-27b itself is repressed by TGF-β [68], its reconstitution with mimics may be especially pertinent in the context of inflammation and fibrosis.

Together, our findings demonstrate potential utility of manipulating miR-27b levels in cardiovascular disease and cancer and confirm its targets as Dll4/Notch axis, PPARγ and its downstream effectors.

## Conclusions

The main non-surgical therapy for ischemic disease involves mechanical stents to maintain blood flow through the diseased vessels. The use of drug-eluting stents was not yet successful. Several experimental strategies aim to improve vascularization in the lower extremities and in the heart by promoting the formation of collateral vessels. They employ stem cell and gene therapies. In stem cell based approaches, induced pluripotent stem cells or bone marrow monocytes are used to enhance local angiogenesis. Gene therapy approaches to enhance local expression of several key growth factors, including VEGF, bFGF and HGF, are subject to the common limitations of gene therapies. None of these approaches have yet reached clinic. We offer targeting the miR-27b-regulated pathways with miR-27b-based drugs for the treatment of cardiovascular disease. Manipulations of microRNA offer safer and more effective alternative to gene therapy. In addition, local use of oligonucleotide mimics of miR-27b facilitates the recruitment of endogenous bone marrow-derived stem cells to the newly forming vasculature at the target site. Thus we provide an alternative to cell-based cardiovascular therapies.

## Additional file

Additional file 1: **Supplementary materials.**

## Abbreviations

ATCC: America Tissue Type Culture Collection
bFGF: Basic Fibroblast Growth Factor
BMDCs: Bone Marrow Derived Cells
CLI: Critical Limb Ischemia
Col: Collagen
DAPI: 4′,6-diamidine-2-phenyl indole
DLI: Doppler Laser Imaging
Dll4: Delta-Like Ligand 4
DMEM: Dulbecco’s Modified Eagle’s Medium
FBS: Fetal Bovine Serum
FDA: Food and Drug Administration
GFP: Green Fluorescent Protein
HGF: Hepatocyte Growth Factor
HLI: Hind Limb Ischemia
IHC: Immunohistochemistry
IL: Interleukin
IM: Intramuscular
LAD: Left Anterior Descending
LLC: Levis Lung Carcinoma
LPS: Lipopolysaccharide
LV: Left Ventricle
LVEDD: Left Ventricle End-Diastolic Diameter
LVESD: Left Ventricle End Systolic Diameter
MI: Myocardial Infarction
miRNA: MicroRNA
MVD: Microvascular Density
NF-κB: Nuclear Factor Kappa Beta
PAD: Peripheral Arterial Disease
PPAR: Peroxisome Proliferator Activator Receptor
RXR: Retinoid Receptor X
SMA: Smooth Muscle Actin
Sema: Semaphorin
Spry-2: Sprouty-2
TAMs: Tumor-Associated Macrophages
TGF: Tumor Derived Growth Factor
VEGF: Vascular Endothelial Growth Factor
