# Supplementary material for: Therapeutic manipulation of angiogenesis with miR-27b
Source: Vasc Cell. 2015 Jun 24;7:6. doi: 10.1186/s13221-015-0031-1 (PMC4497374; doi:10.1186/s13221-015-0031-1)
Supplement: Supplementary file 1 — Supplementary materials. [file 13221_2015_31_MOESM1_ESM.pdf]

Supplementary materials

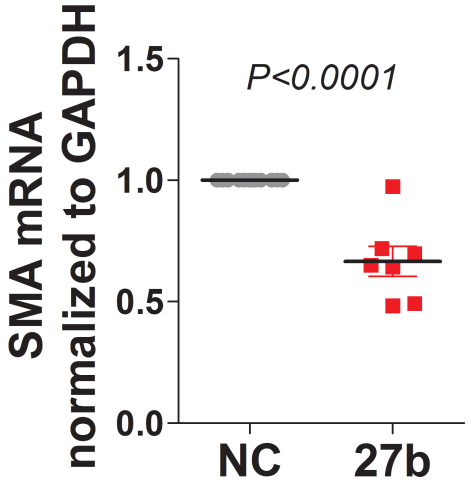

Supplementary Figure 1: Regulation of Smooth Muscle Actin (SMA) by miR-27b in cardiac muscle. Mice subjected to CLI, to induce MI, were treated with negative control (NC) and miR-27b (27b) RNAi mimics by intracardiac injection at the time of surgery. Tissues were harvested on day 28 post-MI. SMA mRNA was measured by real-time RT-PCR and normalized to GAPDH. Data is represented as dot plot, with median values shown. Statistical significance was determined using Wilcoxon non-parametric test.

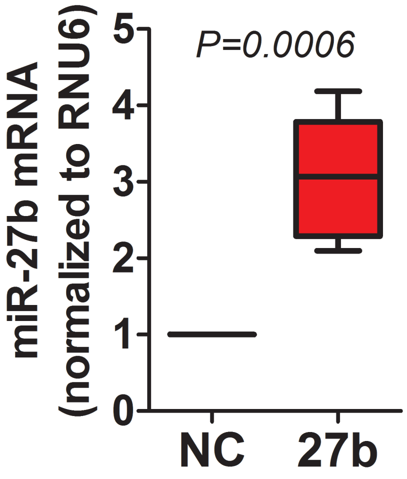

Supplementary Figure 2: miR-27b levels in cardiac tissue. Mice subjected to CLI, to induce MI, were treated with negative control (NC) and miR-27b (27b) RNAi mimics by intracardiac injection at the time of surgery. Tissues were harvested on day 28 post-MI. Mature miR-27b was assessed by real-time RT-PCR and normalized to U6 RNA. Data is represented as box plot with whiskers. Median values are shown. The statistical significance was determined using Wilcoxon non-parametric test.

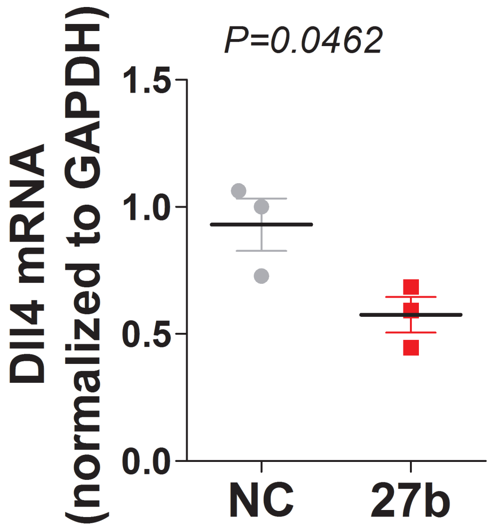

Supplementary Figure 3: Regulation of Delta-Like Ligand 4 (Dll4) by miR-27b in cardiac muscle. Mice subjected to CLI, to induce MI, were treated with negative control (NC) and miR-27b (27b) RNAi mimics by intracardiac injection at the time of surgery. Tissues were harvested on day 28 post-MI. Dll4 mRNA was measured by real-time RT-PCR and normalized to GAPDH. Data is represented as dot plot, with median values shown. Statistical significance was determined using Wilcoxon non-parametric test.

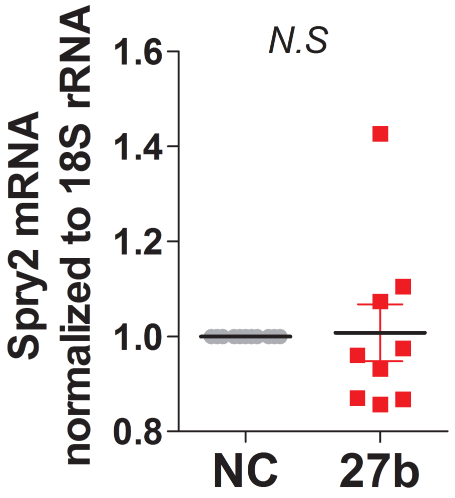

Supplementary Figure 4: Regulation of Sprouty-2 (Spry2) by miR-27b in cardiac muscle. Mice subjected to CLI, to induce MI, were treated with negative control (NC) and miR-27b (27b) RNAi mimics by intracardiac injection at the time of surgery. Tissues were harvested on day 28 post-MI. Spry2 mRNA was measured by real-time RT-PCR and normalized to 18S rRNA. Data is represented as dot plot, with median values shown. Statistical significance was determined using Wilcoxon non-parametric test.

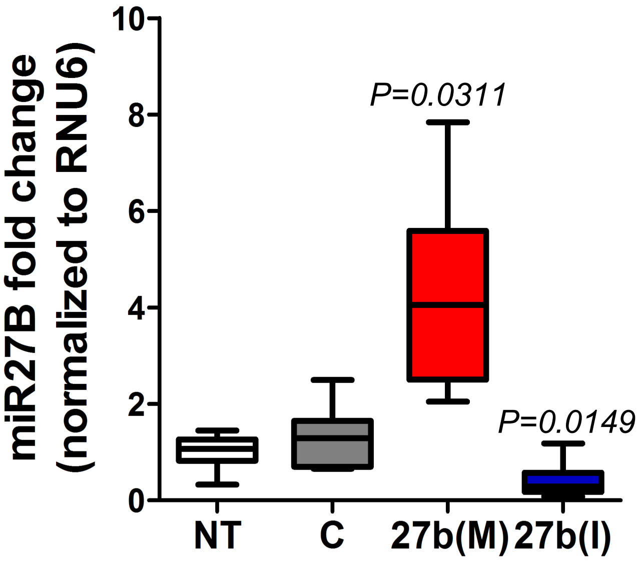

Supplementary Figure 5: miR-27b levels in tumor tissue. Mice were given subcutaneous injections of LLC-1 cells and treated with daily intra-peritoneal injections of control vehicle, negative control and miR-27b RNAi mimic as well as miR-27b inhibitor. At sacrifice, tumors were snap-frozen and miRNA isolated for analysis. Mature miR-27b was assessed by real-time RT-PCR and normalized to U6 nuclear RNA. Data is represented as box plot with whiskers. Median values are shown. The statistical significance was determined using Kruskal-Wallis test.
